# Molecular tools to monitor health and disease – and lucky coincidences

**DOI:** 10.48101/ujms.v127.8987

**Published:** 2022-10-10

**Authors:** Ulf Landegren

**Affiliations:** Department of Immunology, Genetics and Pathology, Science for Life Laboratory, Uppsala University, Uppsala, Sweden

**Keywords:** Padlock probes, proximity ligation, proximity extension, in situ PLA, superRCA, molecular genetics, career, synthetic oligonucleotides, genome project, patent, commercialization

## Abstract

Improved methods for molecular analyses are obviously central for medical research. I will describe herein our work developing tools to reveal molecular states in health and disease. I will recount how I got started in this endeavor, and how our early work characterizing genetic variation led onto high-throughput protein measurements and to techniques for imaging the distribution of proteins and their activity states in tissues. I will also describe a more recent technique to measure even exceedingly rare genetic variants in order to monitor recurrence of disease for tumor patients.

**Figure:**
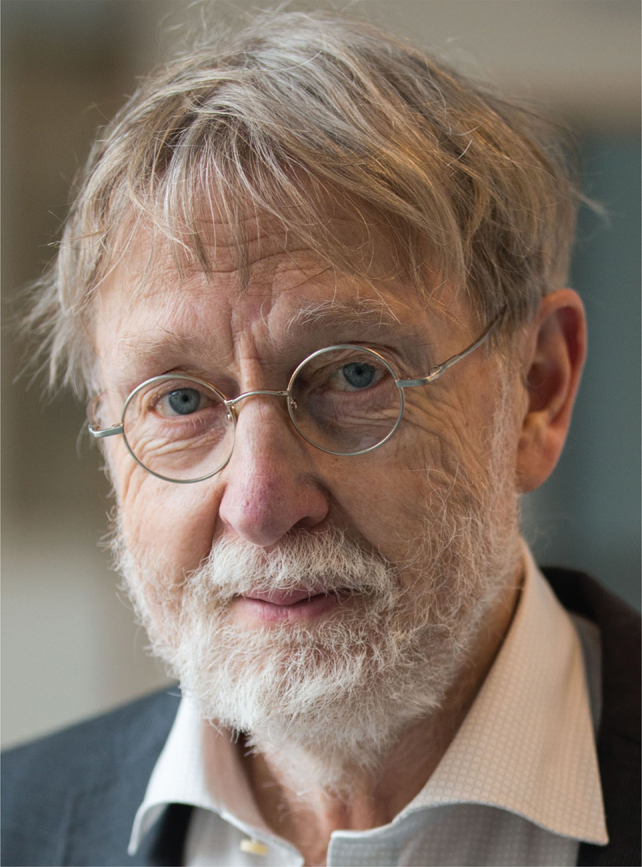
Professor Ulf Landegren, winner of the Medical Faculty of Uppsala University Rudbeck Award 2022. Photo credit: David Naylor.

## Starting out

In relating a chain of events such as a research career, it is tempting to claim a logical overall plan, although this logic may only emerge as an afterthought. As it applies to my career in science, this has surely been, and remains, much of a random walk with stops of variable duration in distinct research fields; more so than for most of my close colleagues, I think. However, it is possible and not entirely dishonest to point to a few guiding principles or inspirations that have dictated a large part of the work that I will briefly summarize herein. I will get back to these principles a little later.

First off, a lucky coincidence gave me my first lab experience. Without any previous contact with research, I happened to land a job at the Swedish University of Agricultural Sciences right after high school. The job was to assist a laboratory assistant. However, there turned out to be no laboratory assistant to assist, and my benevolent boss Associate Prof Gunnar Almgård was more interested in undergrad teaching than in research. Accordingly, at the age of 19, I found myself in a decently equipped albeit windowless lab (it was the anteroom for an animal facility), and with a budget, access to a library and several helpful colleagues. I replicated the work by the subsequent Nobelist Oliver Smithies on isoenzymes as polymorphic markers for genotyping – allowing the distinction of genetic variants. I used the same kinds of markers that Smithies had employed to analyze humans, in order to genotype strains of barley. Altogether I established and applied assays for some 32 markers, and I proudly published my first paper (1).

Realizing that I needed a proper education, I next studied for a medical degree, followed by a PhD in cellular immunology under the stimulating but relatively hands-off Professor Hans Wigzell.

## Experiences at Caltech

In a chance encounter with Professor Lee Hood at a party, he characteristically impressed on me that learning molecular genetics in his lab would be the opportunity of a lifetime, not entirely incorrectly as it would emerge. The suggestion to go to Caltech rather than France suited my wife Christina who did not share my then fascination for French culture. Off we went, with a one-month-old first son in tow, funded by a grant from the Knut and Alice Wallenberg foundation.

I had no prior experience of molecular genetics, but I prepared for the transition by spending the summer of 1984 combining what turned out to be my last clinical rotation in surgery with self-studies of James Watson’s Molecular Biology of the Gene, and in particular the lab manual Molecular Cloning by Maniatis, Sambrook, and Fritsch. The latter became an especially trusted companion, and I used to know by heart the correct page for most techniques I might need.

As a neophyte in molecular genetics, my experience was one of intense, mostly futile struggle, but also great inspiration. The lab at Caltech was overflowing with talented young postdocs, 100 or so in total, many of whom went on to prominent research careers. One of the accomplishments of the Hood lab during my time there, one to which I did not contribute, was the construction of a device to automate readout of Sanger DNA sequencing reactions by monitoring the electrophoretic migration of fluorescent DNA strands past a detector (2). In honesty, the contraption was quite primitive, and this approach to DNA sequencing only became practically useful with further development and commercialization by Applied Biosystems, a company cofounded by Lee Hood. The lab was only too happy to part with the breadboard when the Smithsonian museum in Washington asked to preserve it for posterity among its collections. Nonetheless, this rudimentarily automated sequencing instrument became one of several elements convincing at least some that it might be feasible to sequence the total human genome.

I bet few researchers remember just how controversial the idea was that the human genome could and also ought to be sequenced. The prevailing mindset was that biology is a bewildering infinitude of molecules, processes, and phenotypes, and the thought that we might arrive at complete parts lists of humans - all the genes, transcripts, and proteins - even if feasible, was seen as a big and expensive detour, also conceptually disturbing to many biologists, but inspiring to me.

**Figure 1:**
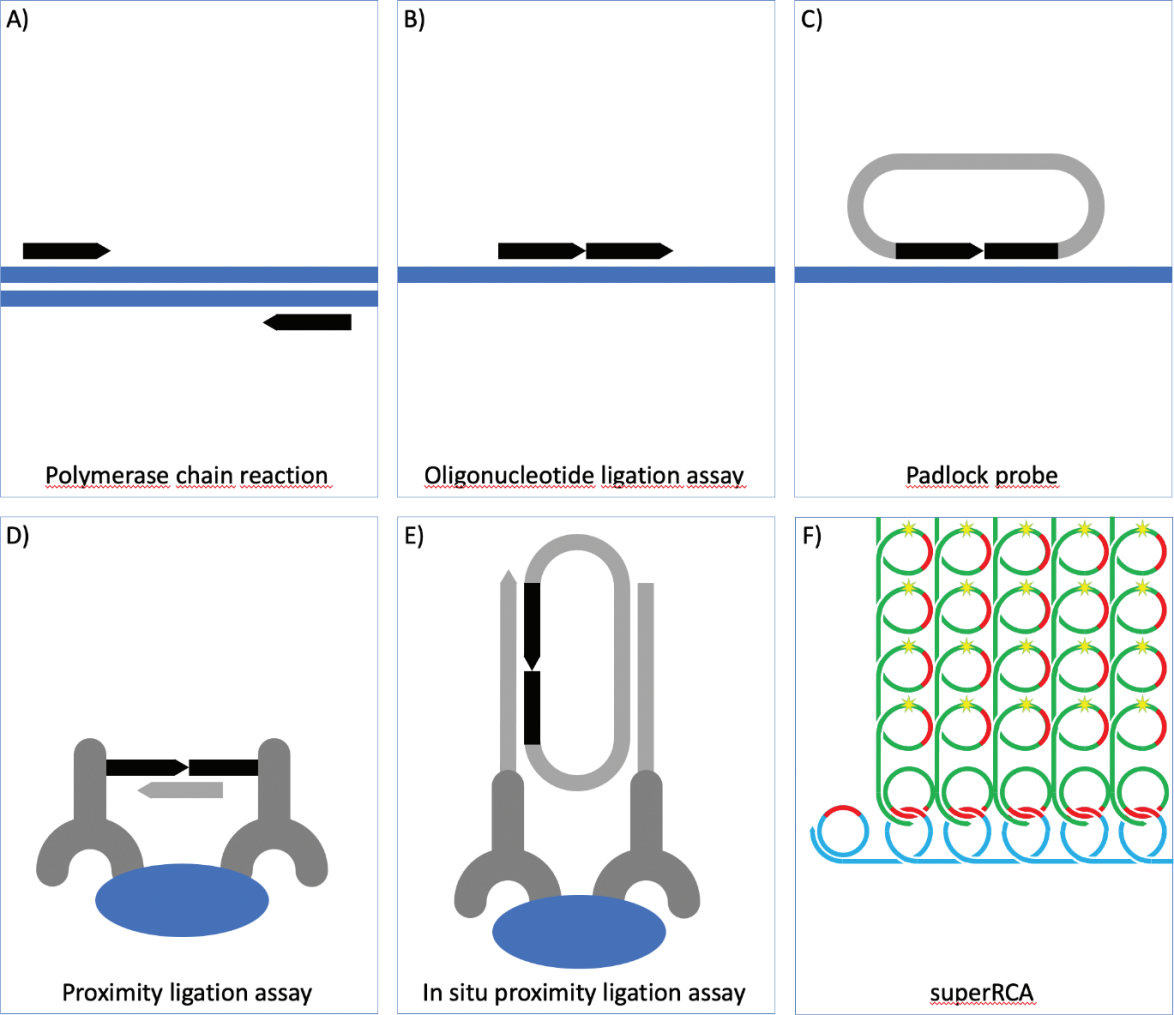
Molecular detection techniques. A) The polymerase chain reaction (PCR) was invented by Kary Mullis who received a Nobel Prize. The technique uses pairs of oligonucleotide primers (black) to exponentially accumulate target sequence copies via iterated polymerization and denaturation reactions. B) In the oligonucleotide ligation assay (OLA) for genotyping, pairs of oligonucleotide probes are joined by ligation, provided that they are correctly matched to their target sequences. C) Padlock probes can be seen as variants of OLA for multiplex genotyping, where the two target-complementary DNA segments are located at either end of longer oligonucleotide probes, such that ligation creates DNA circles. D) In the proximity ligation assay (PLA), target binding by pairs of antibodies gives rise to amplifiable DNA strands by ligation of oligonucleotides conjugated to the antibodies. The assays enable sensitive, multiplex protein detection. E) In situ PLA is a version of PLA where pairwise target binding by oligonucleotide-conjugated antibodies gives rise to circular DNA strands via ligation. These can then be used for localized amplification and detection via rolling-circle amplification (RCA). F) The superRCA reaction can be used for sensitive detection of rare DNA sequence variants. Sequences to be interrogated for the presence of rare mutations are captured in DNA circles. The DNA circles are used to template RCA reactions, and the RCA products are in turn analyzed using padlock probes, specific for mutant or wildtype variants of the DNA sequence. Specifically reacted padlock probes are next in turn subjected to RCA, and the identity and numbers of products of individual starting DNA circles are recorded, for example via standard flow cytometry.

Another major source of inspiration at about the same time came with the publication of the polymerase chain reaction (PCR, 3) (Fig. 1A). Even today, it seems a miracle that oligonucleotide primers can search through complex genomes such as ours in just seconds, copying the proper target sequences with greater than 99.9% efficiency, before repeating this cyclically to exponentially accumulate copies of the target sequence. I realized that given the possibility to design and construct information-carrying synthetic DNA strands at will using simple DNA complementarity rules, and combining these with any of a growing range of purified bacterial enzymes, it is possible to device advanced molecular tools for detection purposes.

## The oligonucleotide ligation assay

With my background in immunology, I was, of course, aware of professor Leif Wide’s famous sandwich immune reaction for protein detection. In this assay, an immobilized antibody captures a target protein for subsequent detection via a second, labeled antibody. I came up with the idea of using pairs of synthetic oligonucleotides, hybridizing to an investigated target DNA sequence, in place of the antibody pairs. By selecting the two oligonucleotide sequences, so that they would base pair to their targets immediately next to each other, I could allow a DNA ligase to join the two hybridized oligonucleotide probes into one longer DNA strand. Moreover, since DNA sequence differences among individuals mostly involve the exchange of single nucleotides, I investigated if by chance the ligase would also help distinguishing between properly matched and mismatched oligonucleotides. This turned out to be the case, and I published this oligonucleotide ligation assay (OLA) as an early genotyping technique (4) (Fig. 1B), but only after the Caltech tech transfer office had helped me write a patent. I was told that this was the most expensive patent written thus far at Caltech. The patent was promptly licensed to Applied Biosystems, and the method was commercialized in quick succession, setting an example for my subsequent work. As is commonly the case, the technique later became subject for conflicts about patent rights and claims of infringement, where I have sometimes served as an expert witness, and I have seen the technique mistakenly referred to as the oligonucleotide litigation assay.

## Inspirations

The Caltech experience left me with some key insights that have guided my subsequent work: 1) It was foreseeable that thanks to the genome project, we would acquire complete molecular parts lists for humans and other organisms. Given suitable means to analyze these features, it would be feasible, at least in principle, to e.g. monitor hereditary components of diseases or assess levels of proteins that reflect states of health, thus vastly increasing opportunities to understand, diagnose, and treat disease. 2) By suitably combining designed synthetic DNA with enzymes, it might be possible to construct the molecular tools needed to analyze this wide variety of molecular features in high throughput. 3) Finally, while academic research is well placed for conceiving and exploring new approaches to molecular detection, the cooperation with industry at later stages can help ensure that these methods are made robust and become widely accessible.

## Return to Sweden

Back in Uppsala in 1989 after almost five years at Caltech, and with another two little boys in the growing family, I was very generously received in Uppsala by professor Ulf Pettersson, who became a most supportive mentor. I hijacked from Ulf my excellent first PhD student Maria Lagerström-Fermér, and via Ulf, I also received valuable starting support by the industrialist Anders Wall through the Kjell and Märta Beijer Foundation.

I continued thinking hard about how to investigate large sets of genetic variants in people in order to trace genetic factors underlying disease. The existence of such factors has long been known in an indirect fashion, for example by comparing disease concordance between mono- and dizygotic twin pairs. With the growing knowledge of common genetic variation in humans, and with sufficiently powerful techniques to genotypes large numbers of such variants in numerous cases and controls, the grounds were being prepared for what some ten years later became known as genome-wide association studies (GWAS) (5, 6), a mature research field by now. There has been some disappointment that these studies have uncovered few genetic markers of diagnostic value. However, the great success of the approach is the valuable insights it has afforded in numerous genetic factors contributing to the risk of developing many different diseases. Some of these factors can point to promising drug targets in the corresponding diseases.

## Padlock probes

To meet what I foresaw as a greatly increased need for high-throughput genotyping techniques, we modified the genotyping OLA reaction. My brilliant PhD student Mats Nilsson demonstrated that reagents could be redesigned for much higher throughput if the two oligonucleotides to be joined by ligation when properly base-paired to the correct target sequence are in fact the two ends of a single, long DNA sequence (7). Upon target recognition, a ligase can join the ends of correctly matched, but not mismatched probes, converting them to DNA circles. Because of the helical nature of duplex DNA molecules, the probes are wound around and therefore topologically linked to their targets. Accordingly, we decided to call them padlock probes (Fig. 1C). Since only intra- but not intermolecular probe ligations give rise to DNA circles, these highly specific probes can be combined in very large numbers (hundreds of thousands in fact), without increasing problems of crossreactivity between different probes. This is in contrast to PCR where multiplexing typically leads to rapidly increasing numbers of non-specific amplification products as more primer pairs are added to a reaction. Large sets of circularized padlock probes can be amplified with a single pair of PCR primers to enhance detection sensitivity, followed by identification of individual reacted probes for genotyping purposes. We later found that yet another interesting property of these circularizing probes is that a suitable DNA polymerase can be employed to copy the “endless” reacted probes. The process, called rolling-circle amplification (RCA), results in a long concatemer of complements of each DNA circle. This reaction presents valuable opportunities for sensitive readout of detection reactions with minimal background, since the RCA reaction is strictly contingent upon proper probe circularization.

**Figure 2:**
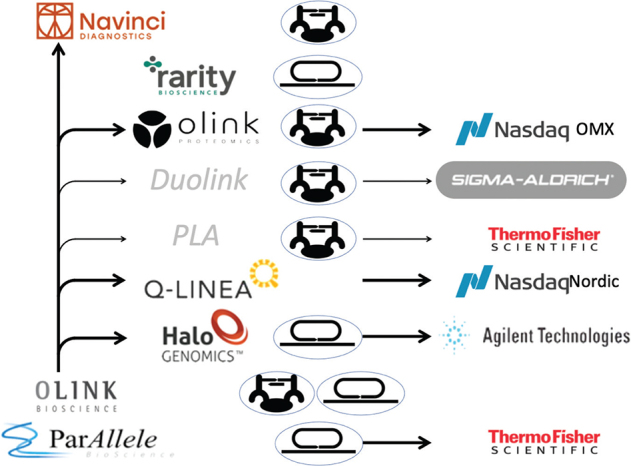
Companies, technologies, exits, and licenses. A compilation of the main companies founded by me and my students, the companies’ main technologies (either padlock probes or PLA), and for some of them exits by trade sales or by entering the stock market. Thin arrows and names in grey italics represent some of the technologies licensed to other companies.

I was eager to further develop and scale up the promising padlock probe technique in a commercial setting as the growing need for high-throughput genotyping was becoming apparent. However, an unfortunate business agreement complicated the founding of such a company in Uppsala. After some delay, Mats and I decided to instead help cofound the company ParAllele in South San Francisco together with colleagues from Stanford. The company successfully used the padlock probe technology to develop high-throughput genotyping assays. Subsequently, a number of other companies have been established by my students and me using variants of the padlock probe concept, and the patent has also been licensed to international companies (Fig. 2). I will get back at the end of this account to a recent application of the basic padlock probe technology.

## Proximity ligation assays

Around the same time that I began thinking seriously about padlock probes in the first half of the 90s, I had the idea that this same DNA ligation mechanism that had served us so well - covalently joining synthetic DNA strands - could perhaps also be used for protein detection assays. If pairs of antibodies jointly binding a target protein molecule were each conjugated to a distinct DNA strand, then their juxtaposition upon pairwise target binding could permit DNA strands to be joined by ligation. If so, then the ligation products could be amplified, identified, and quantified as a measure of the numbers of target protein molecules in the sample in what we termed proximity ligation assays (PLA) (Fig. 1D). In applications for both grants and a patent, I optimistically described this as an ultrasensitive protein assay, but in practice, it took several years before we started getting any useful experimental results. It was further ideas and inspired work by two talented PhD students, Simon Fredriksson and Mats Gullberg, that finally got the method working very well indeed (8).

On the strength of a growing patent portfolio, and with economic resources from the successful exit by ParAllele, some of my PhD students and I started the Uppsala company Olink Bioscience in 2004 together with Björn Ekström as a CEO with ample industrial experience (Fig. 2). Over the years, this company used technology from our lab to award licenses and, in turn, spin out companies. HaloGenomics was founded in 2008, based on research by Mats Nilsson and skillfully led by my former student Olle Ericsson straight from his PhD. The company developed a technology for sample preparation before next-generation sequencing and was acquired by Agilent in 2011. Q-linea (https://www.qlinea.com), also founded in 2008, is marketing a technology for antibiotic susceptibility testing in sepsis developed in house under the creative leadership of my former PhD student Jonas Jarvius. The company is traded at Nasdaq Sweden since 2018. The company Olink Proteomics (https://www.olink.com), spun out in 2016, markets multiplex protein assays. Under the CEO Jon Heimer, the company reached the status of a unicorn as it was valued considerably above a billion dollars by the time it began trading at Nasdaq USA in 2021 (Fig. 2).

## On requirements for detection specificity

This may be a good time for a technical comment - or maybe not if you are uninterested in technology. Anyhow, the PCR technique derives its impressive specificity from the strict requirement that each target sequence must be recognized by two oligonucleotide primers, ignoring any sequences that are only detected by one or the other primer. In a similar manner, padlock probes require that both ends of the probes can hybridize to their target sequences, ensuring a similar degree of specificity of detection in complex DNA samples as that of PCR. As already pointed out, the intramolecular nature of the two target-complementary segments of the padlock probes is key to multiplexing, since ligation reactions that might arise between two different padlock probes fail to yield the DNA circles required for detection and, thus, remain undetected.

In a similar manner to the above DNA detection assays, the classical sandwich immune reactions for protein detection also require target recognition by two antibodies, and again, this has the beneficial effects that any crossreactive binding to a protein by only one or the other antibody in the antibody pair fails to give rise to detection reactions. The PLA technique also depends on dual recognition of target molecules by pairs of antibodies, minimizing crossreactivity. Compared to sandwich immune assays, PLA reactions have the added benefit that the conjugated DNA sequences can be designed so that only cognate pairs of antibody-oligonucleotide conjugates give rise to detectable signals. This property is key for multiplexing, and Olink Proteomics now uses a variant of this technique to routinely analyze thousands of proteins in sample aliquots of a few microliters.

In parenthesis, I note here that the ill-fated US company Theranos famously promised multiplex diagnostic assays in single drops of blood but failed to live up to promises. With the demise of the company, it has become commonplace to conclude that the ambitions of the company were unrealistic. However, applications of padlock probes for nucleic acid analyses and PLA for protein detection both go far beyond what Theranos promised in terms of numbers of DNA and protein analyses that can be performed in small aliquots of patient samples.

## In situ PLA

In 2006, my two postdocs Ola Söderberg and Mats Gullberg skillfully developed an in situ version of PLA (9) (Fig. 1E). In this assay, pairwise binding by oligonucleotide-conjugated antibodies in cell preparations or tissue sections is used to generate DNA circles for detection via RCA. The RCA mechanism serves to generate localized signals far above non-specific background, and individual reaction products can be digitally recorded using standard software for quantitative comparisons. I was mainly focused on that this assay for the first time introduced a requirement for highly specific dual recognition also for localized protein detection assays. However, we quickly found another major advantage of the in situ PLA technique, namely, to reveal interacting or post-translationally modified proteins, by suitably combining pairs of antibodies. This ability to observe protein activity states in situ as reflected in dynamic protein interactions and phosphorylations or other modifications goes beyond the mere in situ assessment of gene expression at the protein level. The assays can yield unique insights in what signaling processes are active in cells and tissues and reflect responses to e.g. drug treatment. Reagents for such analyses were launched by Olink Bioscience as Duolink assay kits, later licensed to Sigma, and these generic assay reagents are now combined with user-selected primary antibodies in thousands of labs worldwide.

## Olink for sale

After developing the PLA technique for solution-phase protein detection as a PhD student in my lab, Simon Fredriksson continued improving the method as a postdoc at Stanford, and later, after joining Olink Bioscience first as CSO and then CEO, he evolved the proximity extension version of the technique (PEA), where the ligase is replaced by a polymerase with similar effect. This version of the technique proved suitable for multiplex, high-throughput protein measurement in solution phase, complementing the in situ protein assays.

In 2011, Olink Bioscience was seven years old, and my cofounders were eager for an exit in order to be rewarded for their hard work. Myself, I was more interested in continuing our work by taking advantage of the growing patent portfolio and the many promising applications. I was encouraged by our successful model of exploring and publishing new technologies and early applications in my academic lab, before some of these perhaps could be transferred to industrial settings either in Olink Bioscience or as further spinouts, or through licensing to other companies.

We received an offer for acquisition of Olink Bioscience at a defined price by an international company. With just a few days to act before I would no longer be in a position to prevent this acquisition, I managed to get in touch with the businessman Bengt Ågerup with the help of professor Olle Kämpe. After some deliberations, Bengt agreed to buy shares of those founders who so wished at the set price, but allowing, or rather demanding, that I remained a shareholder. Because of this fortunate development, we were able to continue the fruitful collaboration between my lab and the company, although I note that such interactions between academics and industry are not always harmonious or based on mutual respect.

The PEA protein panels developed by Simon with coworkers rapidly became a commercial success, and in 2016, we decided to spin out from the mother company Olink Bioscience, Olink Proteomics focusing on solution-phase protein measurements (https://www.olink.com). Olink Proteomics’ assays are now in use globally and by all major pharma companies, allowing sensitive measurement of thousands of proteins in thousands of minimal patient samples, thereby providing new insights in proteins as potential biomarkers in a wide range of diseases and in drug therapy. The high-throughput protein assays can complement GWAS analyses, helping to pinpoint specific genetic factors that influence disease risk, and identifying suitable targets for drug therapy.

Because of the commercial success of the daughter company Olink Proteomics, Olink Bioscience later on had to give up its name to avoid confusion, and this company is now renamed Navinci (https://navinci.se) (Fig. 2). Navinci with CEO Robert Gunnarsson has a timely focus on delivering targeted high-performance localized protein assays using an improved version of the in situ PLA procedure to measure specific proteins and their activity states in patient cells and tissues for improved diagnostics and to monitor responses to therapy.

Over the years, our repertoire of molecular tools has continued expanding, but unlike what was the case for OLA, there is typically a fairly long delay before the methods are ready to be published in the scientific literature. A big part of this has been to wait for the “right” students to come by, and luckily there has been so many “right” PhD students and postdocs! Even after a first publication, a lot of work typically remains before the technology is ready to apply to address an unmet need in an industrial context. My lab is currently working on a small number of new molecular technologies, which, little by little, are approaching the point where they can be published - and where further development could possibly result in new spin-out companies.

## superRCA

I want to end this brief account of our work with molecular technologies by describing a recent addition to the repertoire of molecular tools. Since a number of years, we have been exploring novel approaches for particularly demanding DNA or RNA analyses. Examples of this are when exceedingly rare DNA sequence variants in blood plasma may prove diagnostic for the presence in patients of tumors harboring such mutations. These efforts have met with success due to the creative work by my PhD student and later postdoc Lei Chen, who has established a technique we call superRCA (Fig. 1F). In short, the technique works as follows:

Patient-derived DNA sequences to be evaluated for the possible presence of even very low proportions of specific sequence variants are turned into DNA circles. The DNA circles that form are next replicated by RCA, generating strands that each include up to a thousand copies of the sequences of interest. These sequences are then interrogated with padlock probes specific either for known mutations or for the normal versions of the sequences. Because of the many copies of the target sequence in each amplified strand, the occasional mistyping by the ligase-based probing remains undetectable, as long as the vast majority of the repeated sequences are correctly genotyped. This majority vote-mechanism ensures extremely accurate genotyping.

Once the allele-specific padlock probes have recognized their targets they are converted to DNA circles that are wound around the first RCA products. Then they, in turn, can template secondary RCA reactions. If both consecutive RCA reactions produce strands with 1000 copies from their starting DNA circles, then each detected DNA molecule will yield an object containing up to a million copies of complements of the genotyping padlock probes, either wildtype- or mutant-specific. Each product of this superRCA reaction may thereby encompass 10^8^ nucleotides and have molecular weights of tens of gigaDaltons, highly accurately representing the starting DNA sequences in the sample. Because of their very prominent size, individual products of normal or mutant sequences can be labeled using distinct fluorescent hybridization probes and separately counted using generally available flow cytometers that are normally used for analyzing cells.

Together with our colleagues Lucia Cavelier and Anna Eriksson at Uppsala University Hospital, we applied the superRCA technique to monitor leukemia patients for signs of recurrence of their malignancy (10). We demonstrated specific detection of DNA from as little as one malignant cell among 100 000 normal cells, permitting early detection of recurrence of disease and prompt change of therapy in a manner that fits the routine workflow in clinical genetics and hematology units.

Since tumor patients are increasingly having their tumors sequenced in clinical routine, the superRCA method promises to provide a convenient means to monitor patients for responses to therapy via the presence of tumor-specific mutations. This can either be done among cells in blood and bone marrow for leukemia patients as in our recent paper, or by investigating cell-free DNA in plasma or other bodily fluids from patients with solid tumors. To make this promising technology widely available, we have recently started a company called Rarity Bioscience (https://raritybioscience.com), with CEO Linus Bosaeus (Fig. 2).

## Some form of conclusion

My experience from Caltech came at a time when the ground plans were made for the human genome project, and thus for the accumulation of catalogues of information about the molecular composition of humans and other organisms. I was inspired to start thinking about how to construct molecular tools for large-scale analysis of all these molecular features, taking advantage of in vitro synthesized DNA probes and recombinant enzymes. I have also followed the example of my US host lab in commercializing technologies after first establishing them in an academic setting.

Only later did I realize that back in Uppsala, there was a strong precedent for the fruitful collaboration between academic development of molecular techniques and scale-up and commercialization in industry – the tradition in which my lab now works. The Nobelist professor Arne Tiselius founded the company LKB as well as the biochemistry department at Uppsala University. Over many years, this department enjoyed a highly productive interaction with Pharmacia Fine Chemicals (11). However, by the time I was a PhD student in Uppsala, I did not notice anything of this successful interaction, which by then was essentially over.

Our Molecular Tools research unit at Uppsala University now includes many other group leaders, namely, professor Masood Kamali-Moghaddam, associate professors Xingqi Chen, Lars Forsberg, and Marcel den Hoed, and our recent recruit Dr Daniel Fürth. I am glad that several of them share my interest in establishing molecular technologies.

More generally, I am pleased to see that the ambition to bring research results out of the ivory tower into society is now quite common. I estimate that at present some 800 individuals are working in companies founded by my former students and myself. Many of my academic colleagues now have a similar ambition to see their research applied in industry. I believe an important factor here is the Swedish teacher’s exemption, granting academics in Sweden greater control of their inventions than colleagues in most other countries.

I will say that there is a risk that academy-industry interactions become too much of a norm. Academic research must be allowed to follow its own paths irrespective of whether any industrial applications are in sight, and in most cases that probably is not the case. Nonetheless, it is gratifying that when opportunities for commercial applications can be discerned, there is a growing readiness by university researchers to pursue this, and more support is now available for would-be entrepreneurs. On my part, I am particularly pleased that work from our lab, arrived at through a series of lucky coincidences, may contribute to a greater understanding of the molecular basis of disease and also provide opportunities for new diagnostics and therapies.
